# The Role of Fe(III) in Selective Adsorption of Pullulan on Calcite Surfaces: Experimental Investigation and Molecular Dynamics Simulation

**DOI:** 10.3390/molecules29174194

**Published:** 2024-09-04

**Authors:** Kaiwei Ding, Tingsheng Qiu, Xianhui Qiu, Guanfei Zhao, Qinghao Jiao, Jiangjie Fang, Ruisen Lai, Wenhui Yang

**Affiliations:** 1 College of Resource and Environmental Engineering, Jiangxi University of Science and Technology, Ganzhou 341000, China; dkwjxust@163.com (K.D.); qiutingsheng@163.com (T.Q.); fly.guan@163.com (G.Z.); jiaoqinghao1228@163.com (Q.J.); m18870885685@163.com (R.L.); yangwh233@163.com (W.Y.); 2 Jiangxi Provincial Key Laboratory of Low-Carbon Processing and Utilization of Strategic Metal Mineral Resources, Ganzhou 341000, China; 3 School of Civil and Resources Engineering, University of Science and Technology Beijing, Beijing 100083, China; fang18270287388@xs.ustb.edu.cn

**Keywords:** calcite, fluorite, flotation, pullulan, adsorption, MD

## Abstract

The floatability of fluorite and calcite exhibit similar properties, rendering their flotation separation challenging. Macromolecular polysaccharide reagents containing the polyhydroxyl group have shown broad promising application. The selectivity of polysaccharide is relatively low. In this study, the introduction of Fe^3+^ was employed to enhance the selective adsorption capacity of Pullulan polysaccharide towards fluorite and calcite minerals, thereby achieving effective flotation separation. Furthermore, the mechanism underlying intramolecular interactions was elucidated. The DFT calculation and XPS analysis revealed that the adsorption of Fe^3+^ on the calcite surface was more favorable, leading to the formation of a Ca-O-Fe structure. The MD simulation, XPS analysis, and Zeta potential analysis revealed that the Fe-OH groups on the surface of calcite reacted with the -OH groups in Pullulan and formed bonds, resulting in the formation of a Calcite-Fe-Pullulan structure. This facilitated the attachment of a significant number of Pullulan molecules to the calcite surface. The formation of a hydrophilic layer on the outer surface of calcite by Pullulan, in contrast to the absence of such layer on fluorite’s surface, results in an increased disparity in surface floatability between these two minerals, thereby enhancing the efficiency of flotation separation.

## 1. Introduction

Calcium fluoride (CaF_2_), the main component of fluorite, serves as a crucial non-metallic mineral resource containing fluorine and acts as the core raw material for the fluorine industry. It is widely recognized as a strategic reserve resource in numerous countries [1,2]. The utilization of high-quality fluorite in the missile military industry, nuclear industry, and other sectors is paramount. However, as the development and exploitation of fluorite resources continue to expand, the availability of such premium resources inevitably diminishes [3,4,5]. The current fluorite resources are rarely found in the form of pure CaF_2_ and typically contain impurities such as other minerals and elements. In nature, fluorite often coexists with gangue minerals that have similar elemental (Ca) components, including dolomite, barite, and calcite [6]. The similarity in their floatability makes it challenging to separate and purify them [7].

The flotation recovery of fluorite commonly employs fatty acid reagents as collectors; however, these collectors exhibit limited selectivity towards other calcium-containing minerals such as calcite [8,9]. Thus, it is necessary to add depressants [10,11]. In the past few decades, numerous novel depressants such as sodium silicate, tannic acid, citric acid, polyaspartate, and psyllium gum have been developed and utilized as highly effective calcite depressants [12,13,14,15]. The use of these depressants can also give rise to various adverse effects, including excessive consumption, residues difficult to dispose of, environmental contamination, and other associated issues [12]. In recent years, numerous scholars have conducted extensive research on modified depressants of sodium silicate and metal salts, aiming to achieve enhanced flotation separation efficiency for fluorite. The performance of inorganic depressants, however, exhibits significant fluctuations in actual production due to numerous uncontrollable factors. Furthermore, the accumulation and discharge of inorganic reagent residues have inflicted substantial environmental damage.

The advantage of organic depressants lies in their superior effectiveness and reduced dosage compared to conventional inorganic depressants. The depressant properties of small organic molecules, such as citric acid [16], tartaric acid, oxalic acid, and others, have been extensively studied in relation to calcite depression. However, the limited number of hydrophilic polar groups present in these depressants often results in weak depressed effects [17]. Chen et al. [18] employed a specific combination of dextrin and sodium dodecyl sulfonate (SLS) to selectively separate fluorite and calcite. Dextrin, due to its abundant -OH groups, can form a stable chemisorption with the active site Ca on the surface of calcite, thereby enabling selective depression of calcite. In contrast, dextrin offers advantages such as higher solubility, lower cost, and greater environmental friendliness compared to traditional reagents. Yet, the hydrolysates of the macro-molecular with good solubility exhibit predominantly physical adsorption on the mineral surface, with only a minor fraction undergoing chemical adsorption, resulting in suboptimal depression efficacy. Consequently, they are frequently used in combination with other entities (metal ions) to achieve a stronger depressed effect [19]. Sun et al. [20]. demonstrated through flotation experiments that the combination of Cu^2+^ and soluble starch (SS) exhibited enhanced selective depression on calcite compared to SS alone. Furthermore, Cu^2+^ reacted with SS to form metal ion complexes with excellent selectivity on the surface of fluorite, which possessed a higher adsorption capacity than single SS. This increased the difficulty of depressant SS adsorption while promoting collector adsorption. The combination of metal ions and soluble organic macromolecules [21] represents the current development trend in the field of selective depressants for fluorite and calcium-containing gangue.

**Figure 1:**
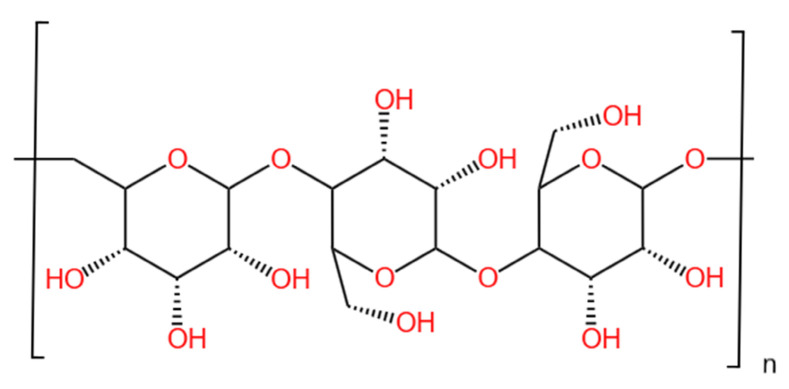
Molecular structure of Pullulan (PU).

Pullulan is a water-soluble mucopolysaccharide produced by microbial exocrine, which is non-toxic and biodegradable. In this paper, PU is used as the abbreviation of Pullulan. PU has been reported as a specific depressant for talc in the flotation separation process of molybdenite and talc [22,23,24]. The presence of numerous hydroxyl groups [25] in Pullulan (PU) can significantly enhance the surface hydrophilicity of minerals when it is adsorbed onto their surfaces. PU, akin to soluble starch (SS), is also capable of forming complexes with metal ions and enhancing their selectivity. Inferentially, PU combined with metal ions may potentially serve as an effective depressant for the flotation separation of fluorite from calcite. Therefore, the impact of water-soluble mucopolysaccharide (Pullulan) and metal ion (Fe^3+^) on the flotation behavior of calcite and fluorite was investigated. The adsorption mechanism of PU and Fe^3+^ onto mineral surfaces was examined through contact angle measurements, Zeta potential, X-ray photoelectron spectroscopy (XPS), and molecular dynamics simulation. The application of biodegradable PU in the flotation separation of fluorite and calcite represents a pioneering effort, offering valuable insights for the eco-friendly recovery of non-metallic minerals like fluorite. The provided Figure 1 illustrates a singular repeat segment of the pullulan polymer molecule.

## 2. Materials and Methods

### 2.1. Samples and Reagents

**Figure 2:**
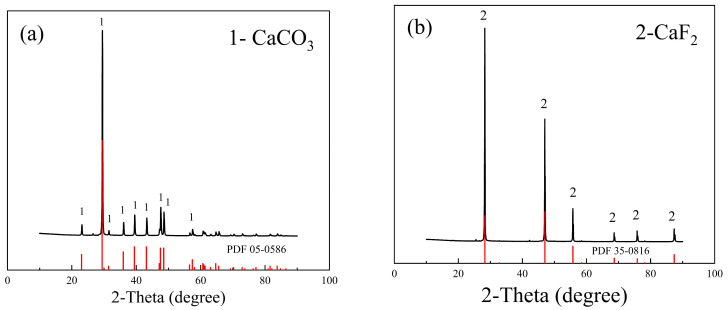
XRD pattern of minerals, (**a**) Calcite; (**b**) Fluorite.

The bulk fluorite and calcite utilized in the experiment were sourced from Inner Mongolia and Hunan, respectively. The bulk ore samples were crushed and grinded, resulting fine mineral samples were screened using Tyler standard sieves and stored sealed. Particles with sizes ranging from 37 to 74 μm were used for flotation experiments and X-ray photoelectron spectroscopy (XPS) tests, while particles with diameters below 37 μm were further pulverized to 8 μm for chemical analysis, X-ray diffraction (XRD) analysis, and Zeta potential measurements. XRD patterns of the samples of calcite and fluorite are depicted in Figure 2. There is no obvious impurity peak in the spectrum, which is in good agreement with the standard reference card of XRD. According to the chemical analysis presented in Table 1, the fluorite sample contains 98.5% CaF_2_, while the calcite sample consists of 99.2% CaCO_3_, high purity meeting the experimental requirements. The collector sodium oleate (NaOL), the depressant PU, and ferric chloride (FeCl_3_) used in the flotation test all meet the purity standard of analysis grade. The pH adjusters are 37% HCl and 99% NaOH. All reagents were purchased from Aladdin Reagent (Shanghai, China). Flotation tests were conducted using ultra-pure water prepared by Haitai Instrument Co., Ltd., Qingdao, China, (TE-S20).

### 2.2. Flotation Experiments

**Figure 3:**
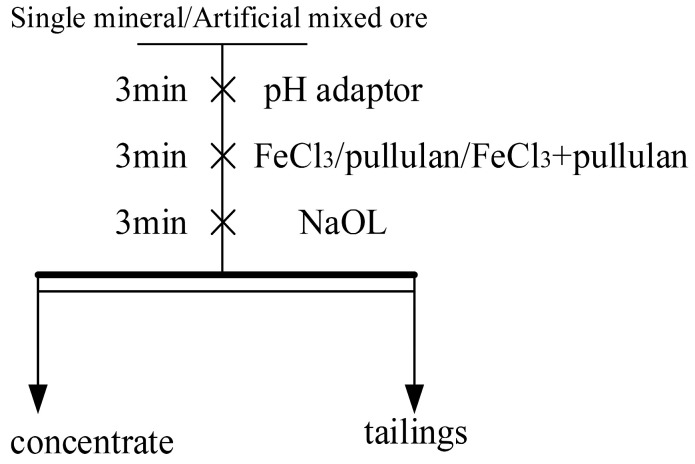
Flotation test flow chart.

The XFGII-type flotation machine was utilized for conducting single-mineral flotation tests, with the stirring speed set at 1850 rpm. The flotation flowchart is shown in Figure 3. Before each experiment, a 2 g sample of single mineral was placed in a beaker filled with water for dispersion and purification using ultrasonic waves for 5 min, aiming to remove any dust and impurities from the surface. Subsequently, the treated mineral sample along with an appropriate amount of deionized water were transferred into a 40 mL flotation cell and stirred for 3 min. The pH of the pulp was adjusted to the desired level by adding HCl or NaOH, followed by sequential addition and stirring of FeCl_3_ reagent, PU reagent, and NaOL reagent for 3 min each. Finally, after collecting the flotation concentrate for 3 min, the obtained concentrated product and the products in the tank were transferred to a vacuum drying oven for thorough drying. Subsequently, the dried product was weighed and its yield and recovery rate were calculated using Equations (1) and (2). Each experimental condition was replicated three times to obtain an average value. The procedure for conducting artificial mixed flotation test remained identical to that of single-mineral flotation test.

Equations (1) and (2) correspond to the calculation formulas of yield and recovery rate, respectively.

γ = [m1/(m1 + m2)] × 100%(1)

ε = [(m1 × β1)/(m1 × β1 + m2 × β2)] × 100%(2)

In the equation, γ is the yield of single-mineral flotation concentrate; ε is the recovery rate of flotation concentrate of artificially mixed mineral (mass ratio of fluorite to galena is 1:1). Among them, m1 is the weight of concentrate and m2 is the weight of tailings. β1 and β2 correspond to the grade of fluorite (calcite) in concentrate and tailings, respectively.

### 2.3. Contact Angle Test

The contact angle was measured using the sessile drop method through the instrument analysis system (JY-82C). The sample with a low impurity content was carefully selected and subjected to polishing using a polishing machine (Tegramin-25, Struers, Denmark) in order to achieve a flawlessly smooth surface, which is essential for conducting precise contact angle testing. The polishing block was then immersed in a beaker containing a ferric chloride solution (FeCl_3_) with a concentration of 20 mg/L and a Pullulan solution (PU) with a concentration of 400 mg/L. After soaking for 30 min, the sample was removed. The residual reagent on the sample surface was rinsed with deionized water and subsequently air-dried. The SL-200C Contact Angle meter (Shanghai, China) was used to measure the contact angle of each sample 3 to 5 times, and the results were averaged.

### 2.4. Zeta Potential Measurement

The Zeta potential measurements were conducted using a 90 Plus PALS Zeta potential analyzer (Brookhaven, GA, USA). A 20 mg sample of −38 μm calcite or fluorite was introduced into a 100 mL beaker containing deionized water and agitated at a constant temperature (25 °C) for 5 min using a magnetic stirrer. The stirred suspension was allowed to stand for 10 min, followed by the addition of 1 mL of the supernatant to a background solution containing 39 mL with a concentration of 1 × 10^−3^ mol/L KCl. The pH value of the solution was adjusted using H_2_SO_4_ and NaOH, followed by addition of the required reagents. The solution was then magnetically stirred for 3 min and left undisturbed for 10 min. Subsequently, the supernatant was collected and transferred to the sample pool for testing. The experiment was conducted with three repetitions for each test, and the mean value was adopted as the ultimate result.

### 2.5. XPS Analysis

X-ray photoelectron spectroscopy was performed using a Thermo K-alpha instrument (Thermo ESCALAB 250XI, Waltham, MA, USA) with a monochromatic Al Kα radiation source of 200 W and an energy of 20 eV. The analysis chamber pressure was maintained at approximately 10^−9^ torr, and the photoelectron detection angle was set to 45° relative to the sample surface. To compensate for surface charge effects, binding energies were referenced to the standard C1s binding energy of 284.8 eV.

### 2.6. Molecular Dynamics Simulation

**Figure 4:**
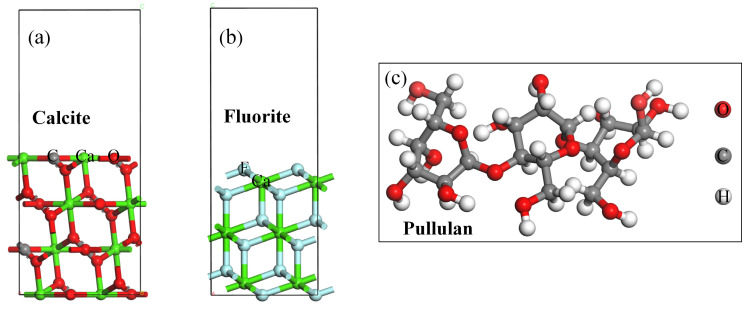
The optimized structures of minerals and reagent, (**a**) Calcite; (**b**) Fluorite; (**c**) Pullulan. (Ca-Green; O—Red; C—Grey; F—Light green; H—White).

The molecular dynamics (MD) calculation was performed using the Forcite module in Materials Studio 2020 software. The geometric structures of mineral crystal cells were optimized using the CASTEP module with appropriate parameters. All crystal cell models were sourced from AMCSD [26], (calcite: 590–600; fluorite: 465–472). Before conducting the MD simulation, the adsorption energy of [Fe(H_2_O)_4_(OH)_2_]^+^ on the surfaces of calcite and fluorite was calculated using castep. Subsequently, the MD simulation was performed utilizing a comprehensive layer–layer model representing the adsorption of [Fe(H_2_O)_4_(OH)_2_]^+^ on calcite. GGA-PBE was employed as the electron exchange correlation functional, while the interaction between valence electrons and nuclei was described by an ultra-soft pseudopotential (USP). The Brillouin region was integrated using a plane wave base set with a 2 × 3 × 1 k grid and a cutoff energy of 480 eV. The system exhibited convergence criteria of 1.0 × 10^−5^ eV/atom, maximum force of 0.03 eV/Å, and maximum atomic displacement of 0.005 Å. For the optimized fluorite and calcite protocells, surface layers {1 1 1} and {1 0 4} were constructed, as shown in Figure 4a,b. The geometrically optimized structure of PU is shown in Figure 4c. The NVE ensemble was employed with three-dimensional periodic boundary conditions for dynamics simulation, while the initial temperature was set to be at 298 K with randomly distributed molecular velocities [27]. The system was then simulated for a duration of 2000 picoseconds, with a time step of 1 fs. A total of 2 million steps were taken, and data were output every 5000 fs. During the simulation, interatomic forces were calculated using the intelligent optimization algorithm within the universal force field module, incorporating Ewald and van der Waals forces.

## 3. Results and Discussion

### 3.1. Flotation Performance

**Figure 5:**
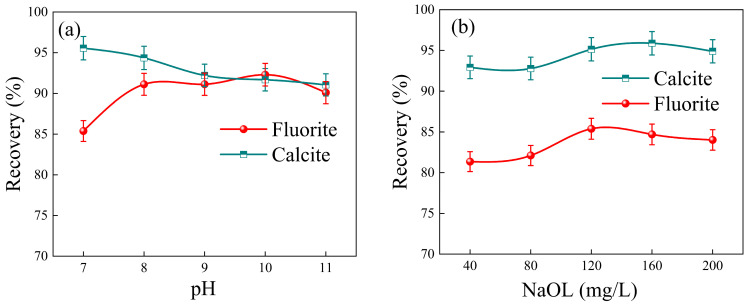

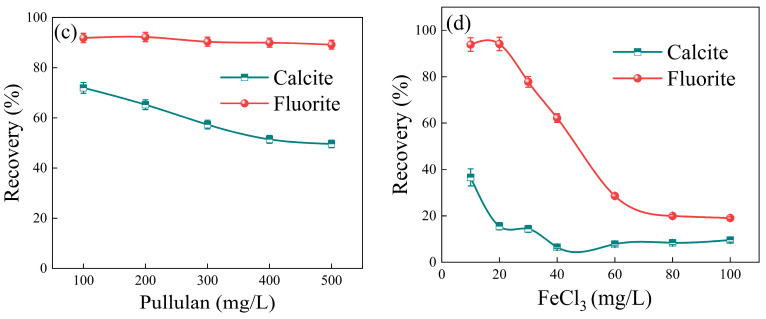
(**a**) The effects of pH on the recovery of fluorite and calcite; (**b**) The effects of collector concentration on the recovery of fluorite and calcite (pH = 7). (**c**) The effects of PU concentration on the recovery of fluorite and calcite (c(NaOL) = 120 mg/L); (**d**) The effects of FeCl_3_ concentration on the recovery of fluorite and calcite (c(NaOL) = 120 mg/L, c(Pullulan) = 400 mg/L).

The flotation process employed NaOL as the collector. The impact of pH on the recovery of fluorite and calcite is demonstrated in Figure 5a. Calcite exhibited a stable recovery rate above 90% within the pH range of 7–12, but its recovery decreased with increasing pH. Fluorite showed an initial increase followed by a decrease in recovery rate with rising pH. At the pH value greater than nine, both minerals exhibited similar recoveries that remained stable at over 90%. Based on these results, a flotation pH value of 7 was selected as optimal.

The impact of NaOL concentration on the recovery of fluorite and calcite at pH = 7 is depicted in Figure 5b. The results demonstrated a clear upward trend in the recoveries of both fluorite and calcite with increasing sodium oleate concentration. When the concentration of NaOL was 120 mg/L, the recoveries of fluorite and calcite were 95.14% and 85.88% respectively. However, at a sodium oleate concentration of 160 mg/L, the recovery rate for fluorite dropped to 86.7%. The above results show that the best recovery rate of fluorite can be obtained when the concentration of collector is 120 mg/L. However, efficient separation between fluorite and calcite was not achievable without the addition of depressants. By introducing PU as a selective depressant for calcite, the difference in flotation performance between fluorite and calcite could be enhanced.

The recovery rate of fluorite remained relatively stable above 90% as the PU concentration increased, as depicted in Figure 5c. Conversely, the recovery rate of calcite gradually decreased with increasing PU concentration. At a PU concentration of 400 mg/L, the recovery rates were 89.95% for fluorite and 51.43% for calcite, representing the largest disparity between the two rates. These results indicated that PU has minimal depressed effects on fluorite while exhibiting some depression towards calcite; however, this depression was not particularly pronounced since the recovery rate of calcite still reaches 50%. The experiments conducted by Sun [20] have demonstrated that the addition of metals can significantly enhance the depressed properties of the depressant. Therefore, in this experiment, the introduction of ferric ion (Fe^3+^) could effectively enhance the selective depression of PU on calcite after careful screening of previous experimental results. As depicted in Figure 5d, the recovery rates of both fluorite and calcite exhibited a declining trend with increasing ferric chloride (FeCl_3_) concentration. Nevertheless, when the FeCl_3_ concentration was below 20 mg/L, the recovery rate of fluorite remained above 95%. In conclusion, effective separation of fluorite and calcite can be achieved when NaOL concentration is set at 120 mg/L, PU at 400 mg/L, and FeCl_3_ at 20 mg/L. Under these conditions, the recovery rate of fluorite reached 94.14%, while that of calcite only amounted to 15.54%. However, when the concentration of Fe^3+^ in solution was high, such as FeCl_3_ concentration exceeding 40 mg/L, calcite also experienced significant depression. This phenomenon could be attributed to the rapid formation of flocculent Fe(OH)_3_ colloids at high Fe^3+^ concentrations, leading to mineral aggregation and precipitation that hindered their flotation into the foam layer. Therefore, a certain concentration of Fe^3+^ and PU combined depressant exerted a strong depressed effect on calcite but had an insignificant impact on fluorite depression. This specific combination enhanced the hydrophilicity of the calcite surface and hindered NaOL adsorption onto it while having minimal influence on NaOL adsorption onto fluorite.

The flotation separation results of artificial mix-minerals of fluorite and calcite are presented in Table 2. Under the conditions of pH = 7, with a collector concentration of 120 mg/L NaOL, a depressant concentration of 400 mg/L PU and 20 mg/L FeCl_3_, and a mass ratio of fluorite to calcite at 1:1, the grade of fluorite reached 85.43% with a recovery rate of 87.21%. It is evident that the utilization of Fe^3+^ and PU achieves favorable outcomes in the flotation separation process between fluorite and calcite when applied at specific concentrations.

### 3.2. Contact Angle Results

**Figure 6:**
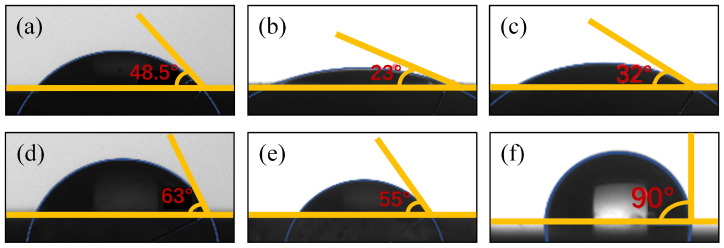
Contact angle of fluorite and calcite surfaces before and after reagent treatment, (**a**) Calcite; (**b**) Calcite + FeCl_3_ + Pullulan; (**c**) Calcite + FeCl_3_ + Pullulan + NaOL; (**d**) Fluorite; (**e**) Fluorite + FeCl_3_ + Pullulan; (**f**) Fluorite + FeCl_3_ + Pullulan + NaOL.

The changes in surface contact angles of fluorite and calcite before and after treatment with FeCl_3_ and PU reagents are illustrated in Figure 6. The exposed surfaces of the two minerals exhibited contact angles of 63° and 48.5°, respectively. Specifically, the contact angle decreased by 25.9° for calcite and by 8° for fluorite upon treatment. These results indicated that Fe^3+^ and PU effectively enhanced the surface hydrophilicity of calcite while exerting minimal influence on that of fluorite. Furthermore, when treated with NaOL after being subjected to Fe^3+^ and PU treatment, the contact angle of calcite only exhibited a slight increase without significant alteration in surface hydrophilicity; conversely, the contact angle on the surface of fluorite increased by 35° following NaOL treatment, leading to a substantial enhancement in its surface hydrophobicity consistent with flotation test outcomes.

### 3.3. Zeta Potential Results

**Figure 7:**
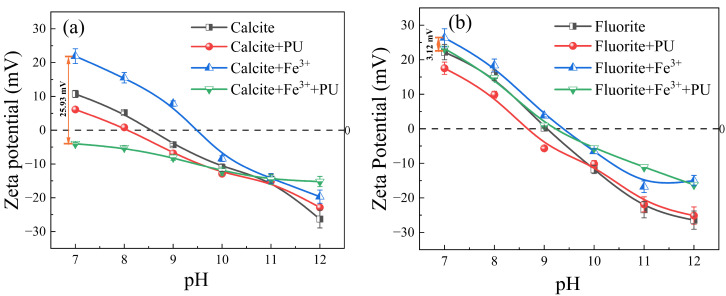
Effects of pH on the Zeta potentials of minerals under different reagents schemes, (c(FeCl_3_) = 20 mg/L, c(Pullulan) = 400 mg/L), (**a**) Calcite; (**b**) Fluorite.

**Figure 8:**
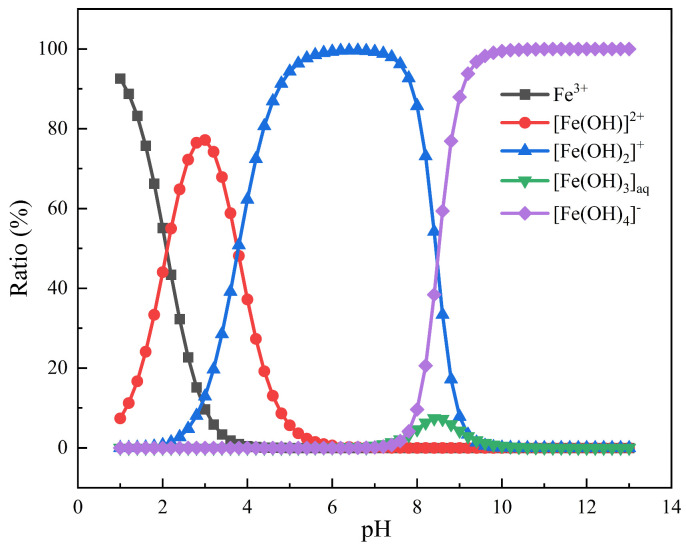
Distribution diagrams of 0.69 mg/L Fe^3+^ (20 mg/L FeCl_3_) solution as a function of pH.

The effects of Pullulan and the addition of Fe^3+^ on the Zeta potential of calcite and fluorite under different pH conditions are illustrated in Figure 7. The isoelectric point of calcite was observed at pH 8.5, while fluorite exhibited an isoelectric point at pH 9. The presence of ionic species at different pH levels, with a concentration of 0.69 mg/L Fe^3+^, is depicted in Figure 8. When the pH is in the range of 4–9, the predominant species of Fe^3+^ is [Fe(OH)_2_]^+^, accompanied by [Fe(OH)]^2+^ and [Fe(OH)_4_]^−^. The formation of Fe(OH)_3_ precipitate at pH > 8 indicated that the potential change was primarily attributed to [Fe(OH)_2_]^+^, [Fe(OH)]^2+^, and [Fe(OH)_4_]^−^. The addition of Fe^3+^ at pH = 7 significantly enhanced the ζ potential value of the calcite surface, whereas there was minimal change in the ζ potential value of the fluorite surface. The FeCl_3_ reagent was introduced into the suspension containing fluorite and calcite, followed by the addition of the Pullulan reagent. The ζ potential of calcite decreased from 21.9 mV to −4.03 mV, and the potential difference reached 25.93 mV, which was much higher than that of fluorite. The addition of the FeCl_3_ reagent could therefore be inferred to promote the selective adsorption of calcite surface by Pullulan, enhance the hydrophilicity of the calcite surface, and inhibit its floating.

### 3.4. X-ray Photoelectron Spectroscopy Analyses

**Figure 9:**
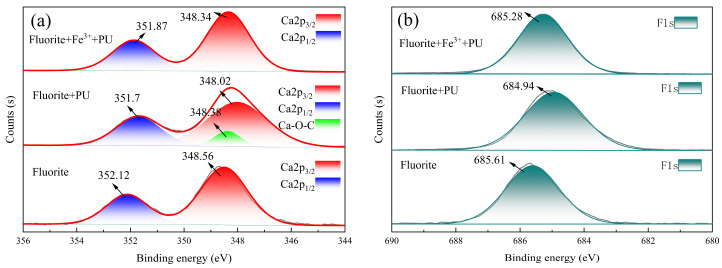
XPS spectra of fluorite and Fe^3+^-treated fluorite before and after Pullulan treatment. (**a**) Ca 2p; (**b**) F 1s.

To investigate the mechanism of the reagent on fluorite and calcite surfaces further, XPS analysis was conducted on samples before and after treatment with the reagent [28]. The obtained XPS spectra were then fitted to obtain fine energy spectra for elements on both minerals’ surfaces in the PU system and the Fe^3+^-PU system. The exposed fluorite surface in Figure 9a exhibited characteristic peaks of Ca 2p3/2 and Ca 2p1/2 at 3448.56 eV and 352.12 eV, respectively. The characteristic peaks of Ca 2p3/2 and Ca 2p1/2 in the PU system exhibited a shift of −0.58 eV and −0.54 eV, respectively, with respect to their original positions [29]. Additionally, a novel peak emerged at 348.38 eV, potentially attributed to the trace adsorption of PU onto the fluorite surface, resulting in the distinctive Ca-O-C characteristic peak. In the Fe^3+^-PU system, the characteristic peaks of Ca-O-C disappeared. The binding energies corresponding to Ca 2p3/2 and Ca 2p1/2 were shifted to 348.34 eV and 351.87 eV, with displacement values of −0.25 eV and −0.22 eV, respectively. The displacement lawed of binding energy corresponding to the characteristic peak of F 1s in Figure 9b was consistent with that observed for Ca 2p3/2 and Ca 2p1/2. The above results demonstrated that the addition of PU induced significant changes in the binding energy of both Ca and F on the surface of fluorite. However, the presence of Fe^3+^ attenuated the impact of PU on the binding energy of elements on fluorite’s surface, suggesting a potential inhibitory effect of Fe^3+^ on PU adsorption onto fluorite.

**Figure 10:**
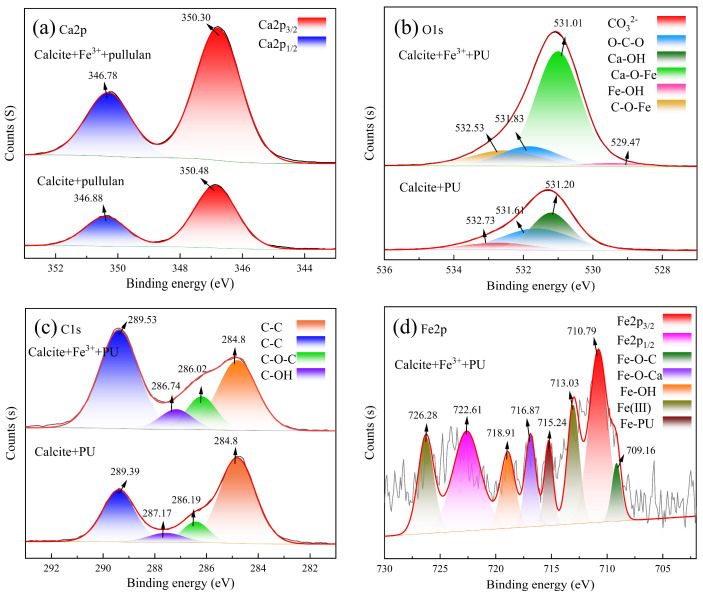
XPS spectra of calcite and Fe^3+^-treated calcite before and after Pullulan treatment. (**a**) Ca 2p; (**b**) O 1s; (**c**) C 1s; (**d**) Fe 3p.

The characteristic peaks of Ca 2p3/2 and Ca 2p1/2 at 346.88 eV and 350.48 eV, respectively, could be observed on the calcite surface of the PU system, as shown in Figure 10a. In the Fe^3+^-PU system, these peaks were shifted to 346.78 eV and 350.30 eV, indicating different chemical states of Ca 2p on the calcite surface due to the addition of Fe^3+^ to the PU system. As depicted in Figure 10b, the surface O 1s spectrum of calcite in the PU system exhibited three distinct characteristic peaks: 531.20 eV, 532.73 eV, and 531.61 eV corresponding to Ca-OH on calcium carbonate’s surface, dissolved CO_3_^2−^ on the surface, and O-C-O in PU, respectively. In contrast, for the Fe^3+^-PU system, there was a shift in the O-C-O peak to 531.83 eV along with a new peak appearing at 529.47 eV, which may represent the Fe-OH characteristic peak similar to Wu’s research findings [30]. The characteristic peak phase corresponding to Ca-OH exhibited a shift towards 531.01 eV in the low-energy direction, giving rise to the formation of a novel characteristic peak of Ca-O-Fe, which could be attributed to the adsorption of Fe^3+^ on the surface of calcite [31]. Additionally, a new peak emerged at 532.53 eV, potentially indicating the distinctive characteristic peak of C-O-Fe in the Fe^3+^-PU complex formation. Figure 10c illustrates the characteristic C-OH peak corresponding to PU in the PU system observed at 287.17 eV, whereas it shifted to 286.74 eV in the Fe^3+^-PU system. This suggests a potential complex reaction between Fe^3+^ and PU.

Figure 10d illustrates Fe 2p3/2 and Fe 2p1/2 exhibiting characteristic peaks at 710.79 eV and 722.61 eV, respectively, and the characteristic peaks of Fe-O-C and Fe-OH observed at 709.16 eV and 718.91 eV, respectively. The characteristic peaks of Fe(III) typically occurred at 713.03 eV and 726.28 eV [32]. The Fe-O-Ca characteristic peak at 716.87 eV was formed by the adsorption of Fe^3+^ on calcite. Through comprehensive analysis of XPS fine spectra of Fe 2p and O 1s, the reaction between Fe^3+^ and PU in the Fe^3+^-PU system led to the formation of a PU-Fe-OH complex with Fe^3+^. This complex then interacted with Ca-OH on the surface of calcite, resulting in the removal of one H_2_O molecule. Ultimately, a stable Ca-O-Fe-PU bond was formed, as PU forms a strong chemical bond with Ca^2+^ on the surface of calcite under the influence of Fe^3+^, leading to stable adsorption.

### 3.5. DFT and MD Simulation Analysis

In order to study the adsorption behavior of Fe^3+^ and PU molecules on mineral surface at atomic scale, MD simulation was performed with PU molecular fragment with a polymerization degree of three. It can be seen from the analysis in Figure 5 that when pH = 7, Fe^3+^ mainly exists in the form of [Fe(OH)_2_]^+^. In order to ensure the accuracy and effectiveness of the simulation, we used [Fe(H_2_O)_4_(OH)_2_]^+^ in the hydrated ion state instead of [Fe(OH)_2_]^+^ for DFT calculation and MD simulation [27,33].

**Figure 11:**
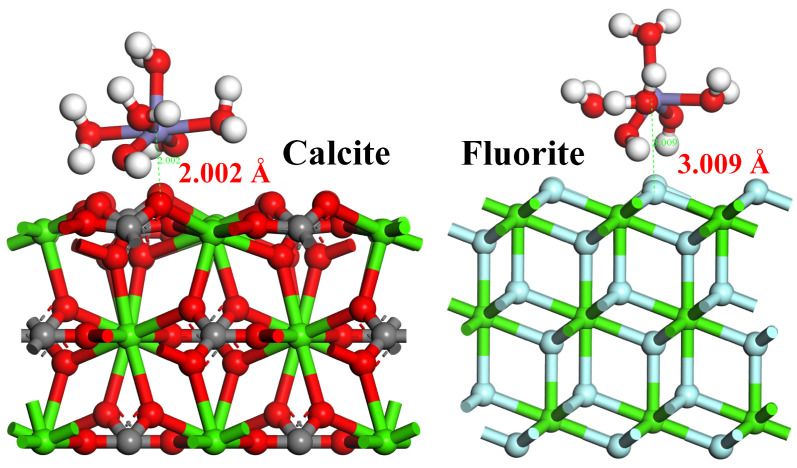
Adsorption configuration of [Fe(H_2_O)_4_(OH)_2_]^+^ on the surface of calcite and fluorite.

The stable adsorption form of [Fe(OH)_2_]^+^ on the surfaces of calcite and fluorite was simulated using the CASTEP module of Material Studio software. The resulting model formed quantum chemistry calculations based on DFT, presented in Figure 11. In the simulation, hydrated iron ions were initially placed 3 Å away from the mineral surface along the *Z*-axis direction. The results demonstrated that [Fe(OH)_2_]^+^ was stably adsorbed onto the calcite surface in the form of [Fe(H_2_O)_4_(OH)_2_]^+^ [34,35], with a reduced spatial distance between the Fe atom and the O atom to 2.002 Å during adsorption. Conversely, Fe atoms exhibited weak repulsion on the fluorite surface, leading to an increased spatial distance between Fe atoms and F atoms to 3.009 Å. Furthermore, these findings indicated that while the adsorption model of [Fe(H_2_O)_4_(OH)_2_]^+^ on the calcite surface was reasonable, it was not suitable for describing its adsorption behavior on the fluorite surface. Therefore, [Fe(H_2_O)_4_(OH)_2_]^+^ only formed stable chemisorption on calcite surfaces, with almost no significant adsorption occurring on fluorite surfaces.

The following equation could be utilized to compute the interaction energy of [Fe(H_2_O)_4_(OH)_2_]^+^, calcite, and fluorite surfaces, thereby facilitating an analysis of the feasibility and intensity of adsorption,

E(ads) = E(Total) − E(Minerals) − E(Hydrated iron ion)(3)

where Ε(ads) represents the adsorption energy, Ε(Total) represents the total energy after [Fe(H_2_O)_4_(OH)_2_]^+^ is adsorbed on the mineral surface, and Ε(Minerals) and Ε(Hydrated iron ion) represent the energy of calcite or fluorite and the energy of [Fe(H_2_O)_4_(OH)_2_]^+^, respectively.

As presented in Table 3, the adsorption energy between calcite and [Fe(H_2_O)_4_(OH)_2_]^+^ was −102.8358 kJ/mol, exhibiting a more negative value compared to the adsorption energy between fluorite and [Fe(H_2_O)_4_(OH)_2_]^+^, which was −1.1718 kJ/mol. It was widely acknowledged in the industry that a more negative adsorption energy indicated greater stability of reagent–mineral surface interactions and stronger adsorption capacity. Therefore, it could be inferred that [Fe(H_2_O)_4_(OH)_2_]^+^ exhibits a higher affinity towards adsorption on the surface of calcite.

**Figure 12:**
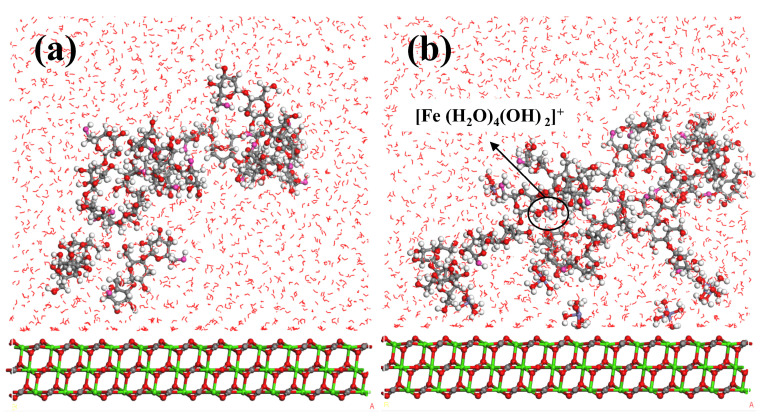
Model of PU adsorption on calcite surface, (**a**) without [Fe(H_2_O)_4_(OH)_2_]^+^; (**b**) covered by [Fe(H_2_O)_4_(OH)_2_]^+^.

**Figure 13:**
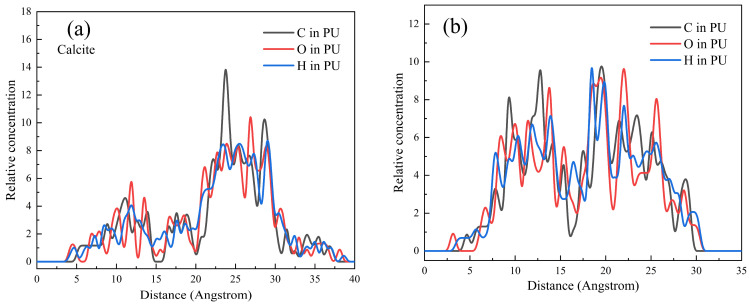
Relative concentration distributions of PU on the surface of calcite along the *Z*-axis, (**a**) without [Fe(H_2_O)_4_(OH)_2_]^+^; (**b**) covered by [Fe(H_2_O)_4_(OH)_2_]^+^.

The microscopic state and mechanism of [Fe(H_2_O)_4_(OH)_2_]^+^ in promoting the selective adsorption of Pullulan on the calcite surface could be investigated through molecular dynamics simulation. A structural model was constructed, depicting an appropriate amount of [Fe(H_2_O)_4_(OH)_2_]^+^ adsorbed onto the surface of calcite. The molecular dynamics model of PU molecule adsorption on the calcite surface is illustrated in Figure 12. The random distribution and diffusion of PU molecules in the absence of [Fe(H_2_O)_4_(OH)_2_]^+^ are illustrated in Figure 12a. In contrast, Figure 12b demonstrates a distinct trend of PU molecule movement and adsorption to the calcite surface when [Fe(H_2_O)_4_(OH)_2_]^+^ is pre-adsorbed on the calcite surface. The presence of [Fe(H_2_O)_4_(OH)_2_]^+^ clearly facilitates the movement and adsorption of PU molecules on the surface of calcite. The concentration distribution of PU molecules is depicted in Figure 13 under both experimental conditions. The distribution of PU on the calcite surface along the Z axis primarily falls within the range of 25~30 Å, as depicted in Figure 13a, in the absence of iron ions. Under these conditions, adsorption of PU onto the calcite surface is minimal, with a majority diffusing into the solution. The relative concentration of PU in Figure 13b exhibits a significant increase within the range of 5–15 Å compared to Figure 13a. The analysis in Figure 12b and Figure 13b suggests that a small quantity of [Fe(H_2_O)_4_(OH)_2_]^+^ diffused into the solution, underwent dehydration with one or more PU molecules to form a complex [36,37,38], thereby facilitating the interaction between PU molecules and Fe. The [Fe(H_2_O)_4_(OH)_2_]^+^ complex acts as a bridging agent, facilitating the adhesion of PU molecules to the calcite surface and enhancing its hydrophilicity.

## 4. Discussion

**Figure 14:**
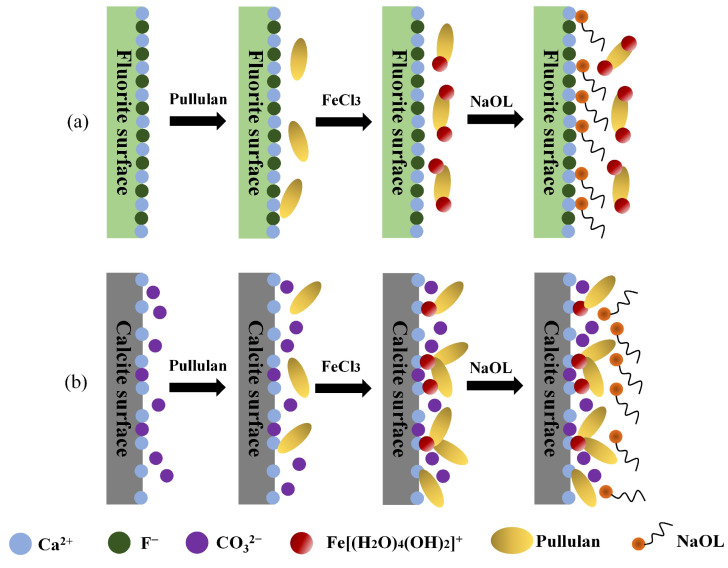
Adsorption model of reagent on the surface of fluorite and calcite. (**a**) Adsorption process of reagent molecules on fluorite surface; (**b**) Adsorption process of reagent molecules on the surface of calcite.

Figure 14 illustrates the schematic diagram depicting the adsorption mechanism model of the reagent on fluorite and calcite surfaces when employing a Fe^3+^-PU composite depressant scheme along with NaOL as a collector. The experiments proved that the addition of Fe^3+^ could enhance the selectivity of PU in the adsorption process. This phenomenon could be attributed to the atomic arrangement on mineral surfaces and molecular structure’s adsorption groups within the reagent. The {111} fluorite and {104} calcite surfaces are widely acknowledged as the most stable cleavage planes for these two minerals [39,40]. The cleavage surface of fluorite exhibits an alternating arrangement of Ca^2+^ and F^−^, as depicted in Figure 14a. A strong electrostatic attraction and chemical affinity between Ca^2+^ and F^−^ hinder the dissolution and detachment of F^−^ from the fluorite surface [41]. The dissociation between carbonate groups occurs on the calcite surface in Figure 14b. The interaction force between Ca^2+^ and CO_3_^2−^ groups was weaker compared to that among the F^−^ and Ca^2+^ groups, allowing for hydrate dissolution of the CO_3_^2−^ groups and complete exposure of Ca^2+^ on the calcite surface. Consequently, the negatively charged reagent PU is more likely to undergo weak physical adsorption on the surface of calcite.

When PU and FeCl_3_ were simultaneously added, Fe^3+^ in FeCl_3_ formed [Fe(H_2_O)_4_(OH)_2_]^+^ in the solution and subsequently complexed with PU. Furthermore, -OH groups and Fe-O- in the Fe^3+^-PU complex also coordinated with Ca^2+^ on the calcite surface, facilitating selective and stable adsorption of PU onto the calcite surface. The arrangement of Ca^2+^ and F^−^ on the surface of fluorite was relatively close, with minimal presence of Ca-OH in its natural state. As a result, the Fe^3+^-PU complex could not connect with either Ca^2+^ or F^−^. Fe^3+^ and F^−^ formed a small amount of stable Fe-F, which could not establish coordination bonds with Pullulan. Additionally, there was electrostatic repulsion between F^−^ on the fluorite surface and the electronegative Fe^3+^-PU complex, preventing coordination bond formation between them. With the assistance of hydrated iron ions, the calcite surface achieved selective adsorption of PU.

## 5. Conclusions

The flotation separation of fluorite and calcite was investigated using Pullulan (PU) and FeCl_3_ as selective inhibitors, and the underlying mechanism was discussed. The results of the single mineral test indicated that at a NaOL concentration of 120 mg/L, Pullulan concentration of 400 mg/L, and FeCl_3_ concentration of 20 mg/L, the recovery rate for fluorite reached an impressive 94.14%, while the recovery rate for calcite was significantly lower at only 15.54%. This demonstrated the successful achievement of flotation separation. The Zeta potential and contact angle tests demonstrated that Fe^3+^ effectively enhanced the adsorption of Pullulan onto the surface of calcite, thereby improving its surface hydrophilicity. Conversely, the impact on fluorite’s surface hydrophilicity was negligible. The DFT calculation and XPS test revealed that Fe^3+^ selectively adsorbed on the calcite surface as [Fe(H_2_O)_4_(OH)_2_]^+^, forming a Ca-O-Fe structure through adsorption with O in CaCO_3_. The reaction between Fe^3+^ and Pullulan resulted in the formation of a C-O-Fe structure, which served as a linkage connecting Pullulan with calcite to generate Ca-O-Fe-Pullulan. The molecular dynamics simulation further confirmed that the presence of Fe^3+^ effectively enhanced the diffusion and adsorption of Pullulan molecules in solution onto the surface of calcite. The presence of Fe^3+^ promoted the desorption of Pullulan from the fluorite surface, thereby further enhancing the difference in hydrophilicity between calcite and fluorite surfaces, ultimately leading to an improvement in foam flotation separation efficiency.

## Figures and Tables

**Table 1:** Chemical composition of fluorite and calcite (%).

Sample	CaCO_3_	CaF_2_
Calcite	99.2	/
Fluorite	/	98.5

**Table 2:** Separation results of artificially mixed fluorite and calcite (%).

Fluorite: Calcite	Product	Fluorite (CaF_2_)		Fluorite
		Yield (%)	Grade (%)	Recovery (%)
1:1	Concentrate	49.82	85.43	87.21
	Products in cell	50.18	12.43	12.79
	Feed	100.00	48.80	100.00

**Table 3:** Adsorption energy of [Fe(H_2_O)_4_(OH)_2_]^+^ with calcite and fluorite surfaces.

Mineral	Surface	Adsorption Energy ∆E/(kJ/mol)
Calcite	{104}	−102.8358
Fluorite	{111}	−1.1718

## Data Availability

The data are available upon request.
